# Sensitive detection of measles virus infection in the blood and tissues of humanized mouse by one-step quantitative RT-PCR

**DOI:** 10.3389/fmicb.2013.00298

**Published:** 2013-10-11

**Authors:** Shota Ikeno, Moto-omi Suzuki, Mahmod Muhsen, Masayuki Ishige, Mie Kobayashi-Ishihara, Shinji Ohno, Makoto Takeda, Tetsuo Nakayama, Yuko Morikawa, Kazutaka Terahara, Seiji Okada, Haruko Takeyama, Yasuko Tsunetsugu-Yokota

**Affiliations:** ^1^Department of Immunology, National Institute of Infectious DiseasesTokyo, Japan; ^2^Cooperative Major in Advanced Health Science, Tokyo University of Agriculture and Technology/Waseda University Graduate School of Collaborative Education CurriculumTokyo, Japan; ^3^Department of Life Science and Medical Bioscience, Graduate School of Advanced Science and Engineering, Waseda UniversityTokyo, Japan; ^4^Department of Virology, Faculty of Medicine, Kyushu UniversityFukuoka, Japan; ^5^Department of Virology III, National Institute of Infectious DiseasesTokyo, Japan; ^6^Kitasato Institute for Life Science, Kitasato UniversityTokyo, Japan; ^7^Division of Hematopoiesis, Center for AIDS Research, Kumamoto UniversityKumamoto, Japan

**Keywords:** measles virus infection, humanized mouse, quantitative RT-PCR, EGFP expression, flow cytometry

## Abstract

Live attenuated measles virus (MV) has long been recognized as a safe and effective vaccine, and it has served as the basis for development of various MV-based vaccines. However, because MV is a human-tropic virus, the evaluation of MV-based vaccines has been hampered by the lack of a small-animal model. The humanized mouse, a recently developed system in which an immunodeficient mouse is transplanted with human fetal tissues or hematopoietic stem cells, may represent a suitable model. Here, we developed a sensitive one-step quantitative reverse transcription (qRT)-PCR that simultaneously measures nucleocapsid (N) and human RNase P mRNA levels. The results can be used to monitor MV infection in a humanized mouse model. Using this method, we elucidated the replication kinetics of MV expressing enhanced green fluorescent protein both *in vitro *and in humanized mice in parallel with flow-cytometric analysis. Because our qRT-PCR system was sensitive enough to detect MV expression using RNA extracted from a small number of cells, it can be used to monitor MV infection in humanized mice by sequential blood sampling.

## INTRODUCTION

Measles, a highly contagious childhood disease caused by the measles virus (MV), affects more than 20 million people each year. MV infection is characterized by a high fever with typical Koplik’s spots followed by the appearance of a generalized maculopapular rash, and is often associated with respiratory and neuronal complications (Griffin, 2007). Since the implementation of vaccination programs using an effective live attenuated MV vaccine, global measles deaths have decreased dramatically. Nevertheless, measles is still one of the leading causes of death among young children under the age of 5 years, especially in countries with weak health infrastructures, and approximately 158,000 measles death occurred in 2011 (). The ongoing global vaccination strategy aims to protect small children at high risk.

The MV vaccine is safe, effective, and inexpensive. Based on its long and successful vaccination history, several groups have taken advantage of reverse-genetics technology to utilize the live attenuated MV vaccine strain as a viral vector to elicit immune responses against foreign antigens from various pathogens, such as Env or Gag of human immunodeficiency virus (HIV; Lorin et al., 2004; Stebbings et al., 2012), hepatitis B surface (S) antigen (Singh et al., 1999; Reyes-del Valle et al., 2009), fusion protein of respiratory syncytial virus (Sawada et al., 2011), and envelope glycoprotein of West Nile virus (Despres et al., 2005; Brandler et al., 2012). MV is a human-tropic virus that uses CD46, signaling of lymphocyte activation molecule (SLAM, CD150), and the recently identified epithelial-cell receptor nectin-4 (PVRL4, see review in Kato et al., 2012) as receptors. To test the immune response against MV-based recombinant vaccines, both MV receptor-transgenic mice (Singh et al., 1999; Lorin et al., 2004; Despres et al., 2005) and non-human primates have been used as animal models (Reyes-del Valle et al., 2009; Brandler et al., 2012; Stebbings et al., 2012).

Although non-human primates are susceptible to MV, and they develop pathologies similar to those that occur in humans, the expense of using monkeys in research limits the number of animals that can be used for studies. To overcome such practical problems, various types of human MV receptor-transgenic mice expressing CD46 or CD150 have been developed (review in Sellin and Horvat, 2009). Unfortunately, MV infection of all of these human MV receptor-expressing mouse models is severely restricted by the presence of murine type I IFN; to establish MV infection, it is necessary to introduce the IFNα receptor knockout into the MV receptor-transgenic mice, even in strains expressing CD150 driven by a native human promoter (Ohno et al., 2007). The IFNα receptor knockout/CD150 knock-in mouse is highly susceptible to MV infection and reproduces some aspects of MV infection in humans, including immunosuppression (Koga et al., 2010). This makes it a useful mouse model for study purposes. However, one problem is the lack of an initial innate immune response, which may modify the outcome of MV infection. Thus, the model may not truly reflect the outcome in humans.

In the early 2000s, a series of immunodeficient mice were developed that allow efficient transplantation of human cells or tissues; these systems are collectively termed “humanized mice.” A large number of studies have described the development of human hematopoietic cells and their immunological functions in humanized mice, and technical modifications have been made for the study of various human diseases (Ito et al., 2012). Currently, humanized mouse systems are widely used as alternatives to non-human primate models, especially for the study of human-tropic infectious diseases such as HIV, human T cell leukemia virus (HTLV), dengue virus, HCV, and EB virus (Akkina, 2013). Of the different humanized mice models, the BM/Liver/Thymus transplanted (BLT) mouse, which is transplanted with human fetal liver and thymus tissue in addition to hematopoietic stem cells (HSCs), is recognized as the model that most closely mimics the human immune response (Wege et al., 2008). However, the use of this model is limited, mainly because of the ethical issues surrounding human fetal organs/tissues.

We have recently established an HIV infection model in NOD/SCID/Jak3null (NOJ) mouse transplanted with human cord blood HSCs (Terahara et al., 2013). To study MV infection in humanized NOJ (hNOJ), we infected an MV vaccine strain (AIK-C) expressing enhanced green fluorescent protein (EGFP) into hNOJ and analyzed the MV-infected cells by flow cytometry. The hNOJ mouse is highly susceptible to MV infection; in that study, we observed that GFP^+^ cells were present in systemic lymphoid tissues and bone marrow (BM). Because it is important to assess MV infection kinetics in an animal without sacrificing the infected mouse, we developed a highly sensitive one-step quantitative reverse transcription-PCR (qRT-PCR) system to monitor MV infection in human peripheral blood mononuclear cells (PBMCs) circulating in the blood of humanized mice. In this study, we describe how this monitoring system works and demonstrate that the results obtained reflect the actual frequency of MV-infected cells, as determined by flow cytometry.

## MATERIALS AND METHODS

### CELL FRACTIONATION OF PBMCs

Peripheral blood mononuclear cells were obtained from human blood samples of healthy volunteers. Samples were collected after obtaining the approval of the institutional ethical committee of the National Institute of Infectious Diseases (NIID; No. 350) and written informed consent from each subject. PBMCs were separated by Ficoll–Hypaque density-gradient centrifugation (Lymphosepal; IBL, Gunma, Japan).

To obtain monocyte-derived dendritic cells (MDDCs), monocytes were enriched from PBMCs using CD14 microbeads (Miltenyi Biotec) and cultured in RPMI 1640 supplemented with 10% fetal bovine serum (FBS), 2 mM glutamine, and antibiotics in the presence of interleukin-4 (IL-4) and granulocyte–macrophage colony-stimulating factor (GM-CSF; both 10 ng/ml, from PeproTech Inc., London, UK) for 1 week. T cells were isolated from CD14-negative PBMCs using the Total T Cell Enrichment Kit (STEMCELL technologies, Vancouver, BC, Canada).

### PREPARATION OF RNA

Total RNA was extracted from mouse blood, BM, and spleen of humanized mice, human PBMCs, and Jurkat cells expressing human SLAM (Jurkat/hSLAM) using the RNeasy Mini Kit (QIAGEN, Valencia, CA, USA) or the Total RNA Isolation Mini Kit (Agilent Technologies, Santa Clara, CA, USA).

To prepare a standard of MV RNA, the cDNA encoding measles virus nucleocapsid (N) (MV-N: AB052821) was subcloned into the pBluescript II vector, and then MV-N RNA was produced by *in vitro* RNA transcription using the T7 RiboMAX^TM^ Express Large Scale RNA Production System (Promega, Madison, WI, USA). The RNA product was purified by DNase treatment, followed by phenol–chloroform extraction and ethanol precipitation, according to the protocol supplied by the manufacturer. The final concentration of RNA was measured using an ND-1000 spectrophotometer (Thermo, Waltham, MA, USA).

### PREPARATION OF STANDARD TEMPLATE DNA

To prepare a standard template DNA, cDNAs of human CD45 (hCD45: NG_007730) and RNase P (NM_006413) were synthesized from total RNA of CEM cells by reverse transcription (RT)-PCR using SuperScript III RT/Platinum Taq Mix (Invitrogen, Carlsbad, CA, USA). The products were further amplified by PCR using TaKaRa Ex Taq Hot Start Version (TAKARA, Otsu, Shiga, Japan) for hCD45, or AmpliTaq Gold 360 (Applied Biosystems, Carlsbad, CA, USA) for RNase P. These PCR products of hCD45 and RNase P were subcloned into plasmids using the pGeneBLAzer TOPO TA Expression kit (Invitrogen) and pGEM-T (Easy) Vector Systems (Promega), respectively.

### REAL-TIME RT-PCR ASSAY

To perform real-time qRT-PCR, SuperScript III Platinum One-Step Quantitative RT-PCR system (Invitrogen) was used according to the manufacturer’s instructions. Briefly, each reaction contained 1× reaction mix, ROX reference dye, SuperScript III RT/Platinum TaqMix, 0.2 μM specific primers, and 0.1 μM TaqMan probe. Reactions were performed on an Mx3000P qPCR system (Agilent Technologies). Thermocycling parameters included a RT step at 50°C for 20 min, followed by a DNA polymerase activation step at 95°C for 2 min and 50 PCR cycles (95°C for 20 s, 60°C for 30 s). Threshold cycle (*C*_t_) values were calculated for each reaction; *C*_t_ represents the cycle at which a statistically significant increase in the emission intensity of the reporter relative to the passive reference dye is first detected.

For detection of hCD45 mRNA, the following sequences were used: forward primer, 5′-GGA AGT GCT GCA ATG TGT CAT T-3′; reverse primer; 5′-CTT GAC ATG CAT ACT ATT ATC TGA TGT CA-3′; TaqMan probe; 5′-FAM-ACA ACT AAA AGT GCT CCT CCA AGC CAG GTC T-BHQ1-3′ (Hamaia et al., 2001). For detection of RNase P mRNA: forward primer, 5′- AGA TTT GGA CCT GCG AGC G-3′; reverse primer, 5′-GAG CGG CTG TCT CCA CAA GT-3′; TaqMan probe, 5′-FAM-TTC TGA CCT GAA GGC TCT GCG CG-BHQ1-3′ (Kimberly et al., 2005). For detection of MV-N RNA: forward primer, 5′-CGA TGA CCC TGA CGT TAG CA-3′; reverse primer, 5′-GCG AAG GTA AGG CCA GAT TG-3′; TaqMan probe, 5′-FAM-AGG CTG TTA GAG GTT GTC CAG AGT GAC CAG-BHQ1-3′ (Hummel et al., 2006).

### GENERATION OF HUMANIZED MICE

Humanized NOD/SCID/JAK3null mice were established as described previously (Terahara et al., 2013). In brief, NOJ mice were transplanted with human HSCs (0.5–1 × 10^5^ cells) enriched from human umbilical cord blood cells into the livers of irradiated (1 Gy) newborn mice within 2 days after birth. All mice were maintained under specific pathogen-free conditions in the animal facility at NIID and were treated in accordance with the guidelines issued by the Institutional Animal Care and Committee of NIID.

Human umbilical cord blood was donated by the Tokyo Cord Blood Bank (Tokyo, Japan) after obtaining informed consent. The use of human umbilical cord blood cells was approved by the Institutional Ethical Committees of NIID and the Tokyo Cord Blood Bank. Human HSCs were isolated using the CD133 MicroBeads Kit (Miltenyi Biotec, Bergisch Gladbach, Germany). The purity was approximately 90% as assessed by flow cytometry.

### PREPARATION AND INFECTION OF MV

Recombinant wild-type MV (IC323: AB016162) expressing EGFP (IC323-EGFP; Hashimoto et al., 2002) and a recombinant vaccine strain of MV (AIK-C: S58435) expressing EGFP (AIK-C-EGFP; Fujino et al., 2007) were grown in Vero/hSLAM cells. Virus titers were determined by plaque assay using Vero/hSLAM cells.

Jurkat/hSLAM cells were infected with various doses of MV [multiplicity of infection (MOI) = 0.25, 0.05, and 0.01] by incubation at 37°C for 1 h, washed twice with phosphate buffered saline (PBS), and seeded on 24-well plates. Cells were harvested immediately after washing (time 0) or 6, 12, 18, or 24 h later. The harvested cells were either lysed for RNA extraction or analyzed by flow cytometry.

Humanized NOD/SCID/JAK3null mice were challenged intravenously (i.v.) with different doses [200, 2,000, 10,000, or 20,000 plaque-forming units (pfu)] of AIK-C-EGFP. Peripheral blood was obtained from MV-infected hNOJ mice at 3, 5, 7, 10, 14, and 21 days post-infection (p.i.). In some experiments, MV-infected hNOJ mice were sacrificed at day 7 p.i. At the time of sacrifice, peripheral blood, BM, spleen, and mesenteric lymph nodes (MLNs) were harvested, and red blood cells were lysed in ACK buffer (0.15 M NH_4_Cl, 1 mM KHCO_3_, and 0.1 mM EDTA-2Na; pH 7.2–7.4).

### FLOW-CYTOMETRIC ANALYSIS OF MV-INFECTED CELLS

PE-conjugated anti-human CD150 (A12) and Pacific Blue-conjugated anti-hCD45 (HI30) monoclonal antibodies (mAbs) were purchased from BioLegend Inc. (San Diego, CA, USA). Cells were stained with these mAbs, fixed with 2% formalin/PBS for 15 min at room temperature, washed, and kept at 4°C prior to flow-cytometric analysis. Dead cells were stained with a LIVE/DEAD Fixable Dead Cell Stain Kit (L34957; Invitrogen). Data were collected using a FACScanto (BD Biosciences, San Jose, CA, USA) and analyzed using the FACSDiva (BD Biosciences) or FlowJo (Tree Star, San Carlos, CA, USA) software.

### STATISTICAL ANALYSIS

Non-parametric one-way ANOVA was performed to compare cell type-specific differences in hCD45 and RNase P mRNA expression. Spearman’s rank correlation coefficient test was also performed to compare the level of MV-N expression and frequency of MV-infected cells. Prism ver.5 software (GraphPad Software, San Diego, CA, USA) was used for all analyses. *P* < 0.05 was considered statistically significant.

## RESULTS

### HUMAN-SPECIFIC qRT-PCR SYSTEM FOR THE DETECTION OF MV INFECTION

**FIGURE 1:**
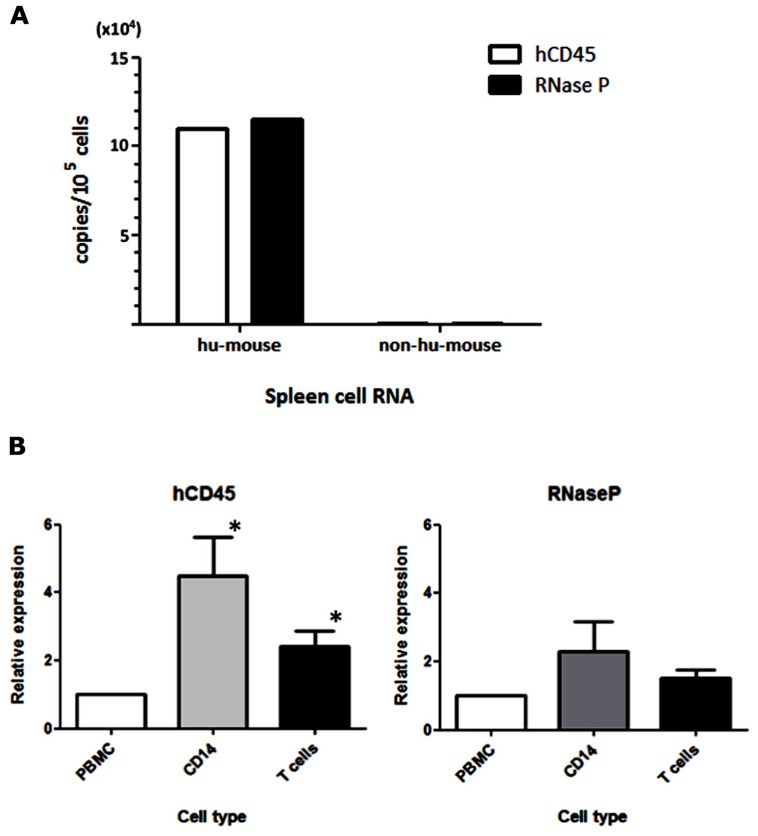
**Selection of an endogenous control for the analysis of MV-infected human PBMCs. (A)** RNA was extracted from spleen cells of hNOJ and non-humanized NOJ, and one-step qRT-PCR was performed using primer and probe sets designed against the human-specific hCD45 and RNaseP mRNAs. To calculate copy numbers of these genes, the PCR products of human CD45 and RNase P were subcloned into plasmids and used as standard DNAs. **(B)** Human PBMCs from five donors were fractionated into CD14^+^ monocytes and T cells. RNA from these cell populations was extracted, and the expression levels of hCD45 and RNase P were analyzed by qRT-PCR. The graph depicts the expression levels in these fractionated cells relative to the levels in PBMCs (defined as 1). Statistical differences in hCD45 and RNase P expression among these cell populations were evaluated by non-parametric one-way ANOVA test (*P<0.05).

For the detection of MV infection in clinical specimens, Hummel et al. (2006) established a sensitive qRT-PCR system that used primer and probe sets targeting the MV-N gene. In our humanized mouse model, it is necessary to analyze endogenous mRNA expression in human PBMCs to determine the level of human cell-associated MV infection in mouse blood. We initially assumed that hCD45 expression would be suitable to discriminate human hematopoietic cells from co-existing mouse hematopoietic cells *in vivo*. On that basis, we designed human-specific primer and TaqMan probe sets for hCD45 and compared their usefulness with a primer/probe set for a widely used housekeeping gene, RNase P. RNA was extracted from humanized (hu-mouse) or non-humanized (non-hu-mouse) murine splenocytes, and the level of mRNA was measured by one-step qRT-PCR. Both hCD45 and RNase P primer/probe sets detected mRNA expression of target genes from human PBMCs present in hu-mouse spleen, at similar sensitivities, but neither set detected expression in non-hu-mouse (**Figure 1A**). Thus, both primer/probe sets are human-specific. Next, we enriched CD14^+^ monocytes and T cells from PBMCs by positive and negative magnetic-bead selection, respectively, and then determined the copy numbers of hCD45 and RNase P in these cell fractions from each of five donors. In **Figure 1B**, the expression levels of hCD45 (left panel) and RNase P (right panel) in monocytes and T cells are depicted relative to the level in each donor’s PBMCs. Because RNase P expression was less affected by cell type than CD45 expression (*P < 0.05), in subsequent experiments we exclusively used RNase P primer/probe sets as an endogenous control for mRNA expression.

### PARALLEL INCREASE IN THE TIME COURSE OF MV-INFECTED CELL FREQUENCY AND MV-N RNA LEVEL* IN VITRO*

**FIGURE 2:**
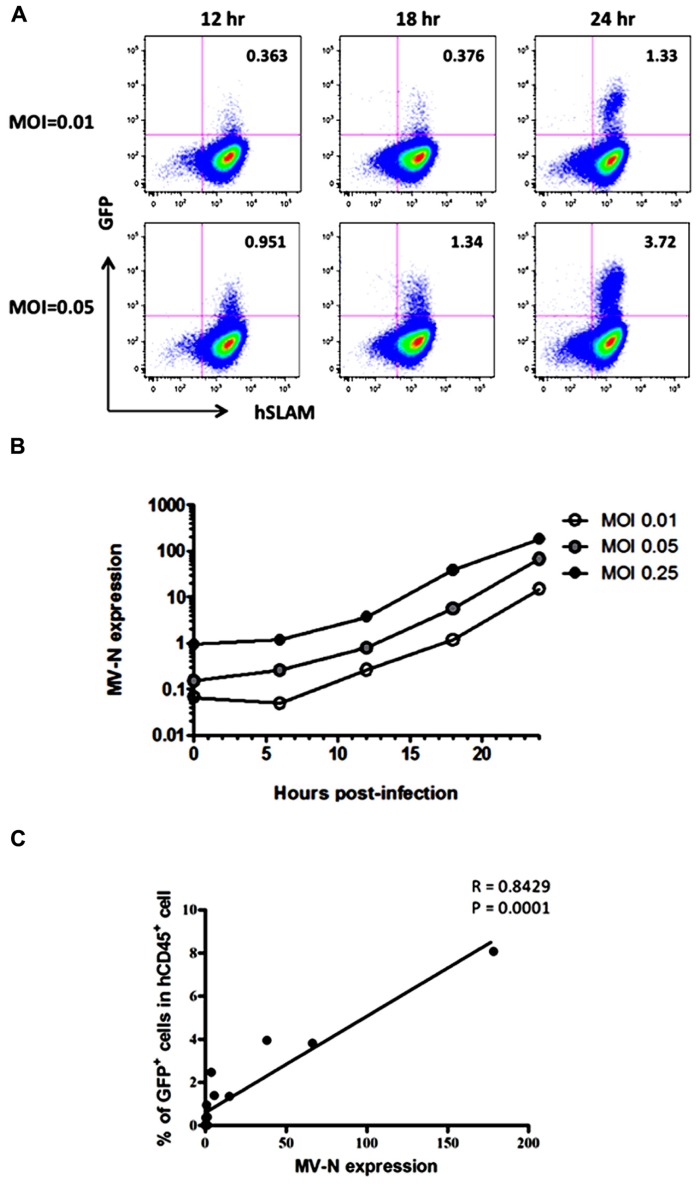
**course of MV infection *in vitro***. Jurkat/hSLAM cells were infected with wild-type MV IC323-EGFP at MOI of 0.01, 0.05, and 0.25, washed, and harvested at the indicated time points. **(A)** Cells were stained with PE-conjugated anti-hSLAM mAb, fixed with 2% formalin/PBS, and GFP expression was analyzed. **(B)** RNA was extracted from cells, and expression levels of MV-N and RNase P were analyzed by one-step qRT-PCR. The copy numbers of MV-N and RNase P were determined, and the ratio of MV-N copies to RNase P copies is depicted on the vertical axis. **(C)** Correlation between the percentage of GFP^+^ Jurkat/SLAM cells and the time course of MV-N expression. Spearman’s rank correlation coefficient was used for statistical analysis.

Because wild-type MV mainly utilizes SLAM as the receptor for entry into lymphoid cells (Tatsuo et al., 2000), the kinetics of MV infection in Jurkat/hSLAM cells can be clearly visualized by flow cytometry. We infected Jurkat/hSLAM cells with a wild-type MV encoding EGFP (IC323-EGFP) at MOI of 0.01, 0.05, and 0.25. Cells were washed and harvested at 6, 12, 18, or 24 h after MV infection. A subset of the cells in each sample was analyzed by flow cytometry, and the remainder of the sample was used for RNA extraction. The mRNA levels of MV-N and RNase P were determined by qRT-PCR, and the level of MV-N mRNA relative to RNase P RNA was calculated. Representative results of three experiments are shown in **Figure 2A** (flow cytometry) and **Figure 2B** (qRT-PCR). Because of the rapid and strong cytopathic effect by MV at the highest MOI (0.25), we omitted the flow cytometry data corresponding to that condition. At MOI 0.01, a similar frequency of GFP^+^ cells was detectable at 12 and 18 h p.i., whereas at MOI 0.05, the GFP^+^ cell frequency was already high at 12 h p.i. Note that the level of hSLAM was not down-modulated by MV infection. Over the time course, relative MV-N expression level at all three MOIs increased in parallel over two orders of magnitude, indicating that these two methods yield comparable results (as shown in **Figure 2C**) and are useful for monitoring the replication kinetics of MV infection* in vitro*.

### PARALLEL INCREASE OF MV-INFECTED CELL FREQUENCY AND MV-N RNA LEVELS* IN VIVO*

**FIGURE 3:**
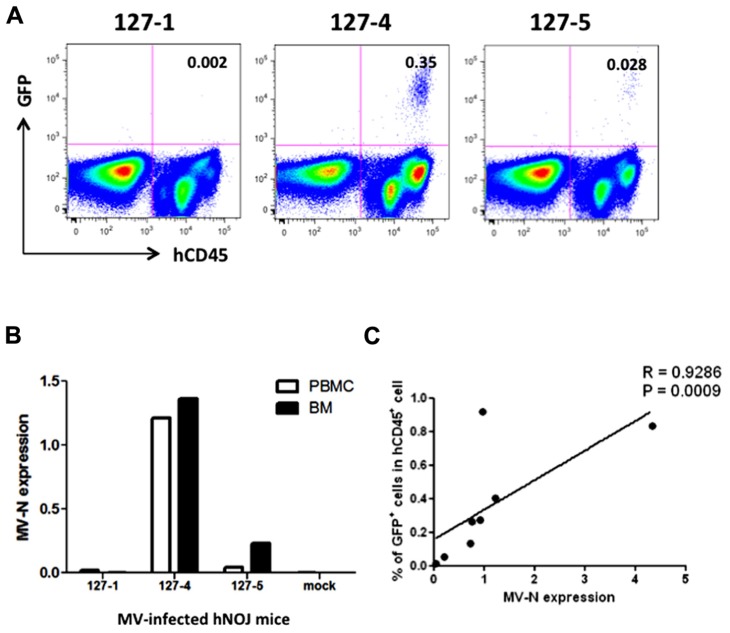
**Analysis of MV infection *in vivo***. Three hNOJ mice (127-1, -4, and -5) were infected intravenously with 2,000 pfu of the MV vaccine strain, AIK-C-EGFP. Mice were sacrificed at day 7 post-infection, and blood and bone marrow cells (BM) were obtained. **(A)** BM cells were stained with PB-anti-human CD45 mAb, fixed with 2% formalin/PBS, and GFP expression was analyzed. **(B)** PBMCs from blood and BM cells were lysed, and RNA was prepared. The expression MV-N and RNase P was analyzed as described in the legend for **Figure 2B**. **(C)** Correlation between the percentage of GFP^+^ cells among hCD45^+^ cells in BM and the level of MV-N expression in MV-infected hNOJ mice, at day 7 (*n* = 4) or day 10 (*n* = 4) p.i. Spearman’s rank correlation coefficient was used for statistical analysis.

We then applied these detection systems *in vivo* in MV-infected hNOJ mice. hNOJ mice were infected with an MV vaccine strain expressing EGFP (AIK-C-EGFP) at 2000 pfu, and the animals were sacrificed 7 days later. Blood PBMCs and BM cells were washed with PBS, and a subset of the cells in each sample were stained with anti-hCD45 mAb. Representative results of flow-cytometric analysis of BM cells from three mice are shown in **Figure 3A**. The percentages of GFP^+^ cells in mice 127-1, 127-4, and 127-5 mice were low (0.002%), high (0.35%), and intermediate (0.028%), respectively. The number of human PBMCs obtained from mouse blood was not sufficient to determine GFP^+^ cell frequencies by flow cytometry. Next, we extracted RNA from PBMCs and BM cells and analyzed MV-N expression by qRT-PCR, as described in the previous section. MV-N expression paralleled the GFP^+^ frequencies in BM (**Figure 3B**). Notably, a high level of MV-N expression was also detected in PBMCs of mouse 127-4, suggesting that the level of MV-N expression per single hematopoietic cell is similar between blood and BM. We plotted the GFP^+^ frequency and MV-N expression level in BM cells of eight mice. As shown in **Figure 3C**, these values were well correlated (*R* = 0.9286). Taken together, these data indicate that MV infection *in vivo* is detectable in BM by both flow cytometry and MV-N RNA qRT-PCR analysis, but only MV-N RNA qRT-PCR is sensitive enough to detect PBMC-associated MV infection in the blood.

### KINETICS OF MV GROWTH CAN BE MONITORED IN THE BLOOD OF hNOJ MOUSE

**FIGURE 4:**
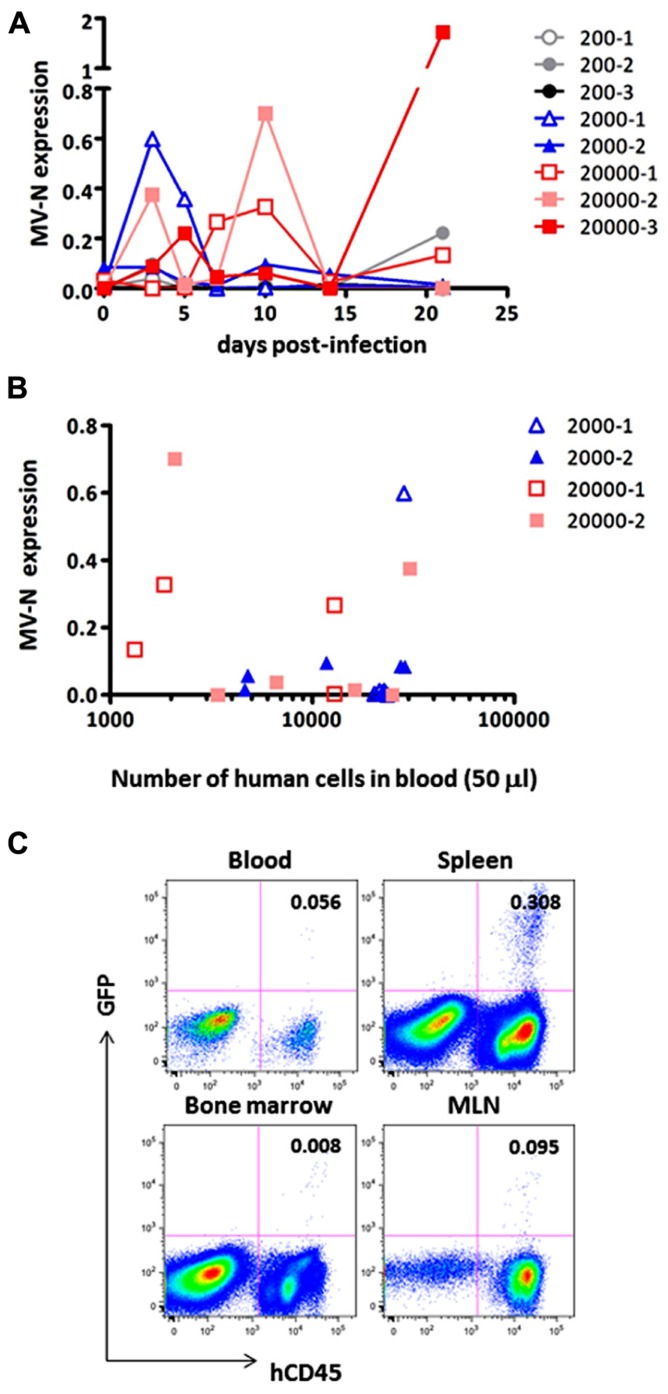
**Monitoring of MV replication *in vivo***. Two to three hNOJ mice per group were challenged with MV AIK-C-EGFP at 20,000 (squares), 2,000 (triangles), or 200 pfu (circles). The PBMCs of these mice were collected at day 3, 5, 7, 10, 14, and 21 p.i. **(A)** The level of MV-N expression in the blood of infected hNOJ. Vertical axis shows the level of MV-N relative to that of RNaseP, as described in the legend for **Figure 2B**. **(B)** For some mice (depicted using the same symbol as **(A)**), the number of human cells per 50 μl of blood used for RNA extraction and analyzed for MV-N expression was plotted on the X-axis. **(C)** An hNOJ mouse infected with MV at a low dose (gray closed circle, 200-2) exhibited an increased level of MV-N. At day 21 p.i., the mouse was sacrificed; cells from blood, spleen, BM, and mesenteric lymph node (MLN) were prepared. Cells were stained and analyzed as described in the legend for **Figure 2A**.

Finally, we measured MV growth kinetics *in vivo* by qRT-PCR analysis using sequential blood samples obtained from MV-infected hNOJ mice; it was not feasible to perform these measurements by flow cytometry because of the paucity of human PBMCs in the blood. Two or three hNOJ mice in each group were infected intravenously with 200, 2000, or 20,000 pfu AIK-C-EGFP and followed up to 21 days p.i. The level of PBMC-associated MV RNA in individual mice is shown in **Figure 4A**. We noticed two peaks of MV replication, the first at around day 3 p.i., and the second at day 10 p.i., irrespective of the initial inoculum. Two mice infected with 20,000 pfu MV exhibited a high level of MV replication that peaked at day 10 p.i. One mouse infected with 2,000 pfu exhibited a high level of MV replication at day 3 p.i., followed by a small peak at day 10 p.i. For some mice, we counted the number of human cells per 50 μl of blood used for RNA extraction. The data are shown in **Figure 4B**. We were able to detect high levels of MV in samples containing less than 2,000 cells, indicating that the qRT-PCR system is sensitive enough to detect low numbers of MV-infected human cells.

Although MV replication was not obvious in three mice infected with the smallest dose (200 pfu), one of these animals exhibited an increase in MV RNA expression at day 21 p.i. (gray circle). We sacrificed this particular mouse and used flow cytometry to analyze GFP expression in its blood, spleen, MLN, and BM. As shown in **Figure 4C**, GFP^+^ cells were present in spleen (0.308%) and all the other tissues, albeit at a lower frequency, indicating that MV infection can occur even at a low dose (200 pfu) and spread slowly in the systemic lymphoid tissues of hNOJ.

It may be necessary to acquire at least 30,000 events to be sure of having >10,000 cells for flow cytometry analysis. This is because of the substantial amount of sample loss that occurs in this system. The flow cytometry data presented in **Figure 4C** were obtained by analyzing ~0.4 ml blood from a sacrificed mouse. However, even under these conditions, the proportion of MV-infected cells detected was only 0.056%; indeed, the cells are barely visible on the plot. Therefore, it appears that flow cytometry is not a suitable method for the sequential monitoring of infected (GFP^+^) cells. Thus, the qRT-PCR system we have developed here allowed us to monitor systemic MV replication using a small volume of blood from humanized mice.

## DISCUSSION

Based on a highly sensitive MV-N RNA detection method previously developed by Hummel et al. (2006), which could detect one copy of synthetic MV RNA/reaction, we developed a novel one-step real-time qRT-PCR system for the purpose of monitoring MV replication in the blood of MV-infected humanized mice. Because MV replication usually occurs in association with cells (Griffin, 2007), it is necessary to evaluate the endogenous RNA expression level of human PBMCs that co-exist with mouse blood cells. To this end, we designed human-specific primer/probe sets for the CD45 and RNase P mRNAs. When we analyzed the detection efficiencies of these two primer/probe sets using distinct cell types present in human PBMCs, we found that RNase P expression was less dependent than CD45 expression on cell type. Using this qRT-PCR system with RNase P as an internal control, we can reliably detect MV replication with high sensitivity in humanized mice *in vivo*. When MV expressing GFP was used for infections *in vitro* or *in vivo*, the level of MV-N RNA was closely correlated with the frequencies of GFP^+^ MV-infected cells determined by flow cytometry.

Our qRT-PCR system allowed us to follow MV replication *in vivo* using a small amount of blood, with no need to sacrifice mice at each time point. Although flow-cytometric analysis provides valuable information, such as the proportions of various cell types and the surface phenotypes of MV-infected cells, the small number of human cells circulating in the mouse blood may not be sufficient for precise estimation of MV-infected cells by flow cytometry. By contrast, our qRT-PCR system was able to detect MV-N RNA in fewer than 2,000 human PBMCs (**Figure 4B**). This is an important technological advantage considering that individual humanized mice exhibit variable levels of human cell engraftment, i.e., chimerism (Terahara et al., 2013); moreover, there may exist donor-to-donor variation in susceptibility to MV infection. Thus, it should be possible to select humanized mice with a degree of MV infection appropriate for the purpose of a given experiment.

In this study, MV was inoculated through the tail vein, and infected cells were distributed to systemic lymphoid tissues as well as BMs, where human hematopoietic cells localize in humanized mice (Traggiai et al., 2004). MV may also be distributed to other organs, such as lung and intestinal tissue, as demonstrated in the case of HIV infection using the BLT mouse (Sun et al., 2007). To our surprise, by monitoring MV replication in PBMCs of humanized mice, we noticed two peaks of MV replication, at around 3 and 10 days p.i., in some mice. This pattern of MV replication did not depend on the initial dose of MV inoculum. We do not know why MV replication showed two peaks in many animals*.* However, it was recently reported in a monkey model that MV RNA persists in PBMCs for more than 1 month after primary infection, and declined in three phases (Lin et al., 2012). The authors of that study hypothesized that both T cells, including regulatory T cells (Treg), and antibody responses contributed to the dynamics of MV replication *in vivo*. Although hNOJ mice are reported to show poor immune responses, the role of regulatory T cells should be considered. This is because these cells regulate HIV-1 infection in humanized mice (Jiang et al., 2008). Alternatively, it may be that the intravenous injection of MV rapidly kills the target cells (probably those showing an activated phenotype) within 3 days. The low number of MV-infected cells then gradually transmits the virus to the human cells that are replenished from the BM stem cell pool. Further investigations are required to clarify this issue.

The humanized mouse model is expected to be a useful tool for studying virus infection (Akkina, 2013). Although the human immune system is not fully reconstructed by the transplantation of human HSCs alone, we believe that further improvements are possible, which will allow us to utilize this mouse model to not only evaluate vaccine and drug efficacy but also to increase our understanding of the pathogenesis of MV infection. The described novel method of monitoring MV-infected human cells in the blood will be useful for studying MV-based vaccines in humanized mouse models without the need to sacrifice the mice.

## Conflict of Interest Statement

The authors declare that the research was conducted in the absence of any commercial or financial relationships that could be construed as a potential conflict of interest.
